# Technology-driven solutions to prompt conversation, aid communication and support interaction for people with dementia and their caregivers: a systematic literature review

**DOI:** 10.1186/s12877-021-02105-0

**Published:** 2021-03-04

**Authors:** Viktoria Hoel, Carine Mendom Feunou, Karin Wolf-Ostermann

**Affiliations:** Institute for Public Health and Nursing Research, Health Sciences Bremen, University of Bremen, Grazer Straße 4, 28359 Bremen, Germany

**Keywords:** Dementia, Informal caregivers, Quality of life, Technology, Social health, Communication, Dyadic relationship

## Abstract

**Background:**

The impact of dementia for communication skills can result in difficulties in social interactions between people with dementia and their conversation partner, as initiating and maintaining conversations becomes increasingly challenging. The role of technology in enhancing social health and participation for people with dementia is increasing, but the use of technological devices to support social interactions and aid communication quality is still in its infancy. The objective of this literature review is to provide a comprehensive description of technology-driven interventions for people with dementia and their conversation partners to prompt communication and facilitate positive social interactions.

**Methods:**

A systematic search was conducted using PubMed, CINAHL and PsycINFO, with titles and abstracts independently screened by two researchers. Quality appraisal of the included studies was assessed using the Mixed Methods Appraisal Tool.

**Results:**

Of the 18 papers included, the technology most commonly used to facilitate communication and interactions was tablet-computers (*n* = 7), social robots (*n* = 5) and computers systems (*n* = 4). By analyzing the impact of the device(s) for social interaction and communication, four major themes emerged: i) breaking the ice; ii) increased interaction; iii) better understanding of the person with dementia; and iv) reduced pressure for the conversation partner.

**Conclusion:**

While the majority of the included studies are small-scale, they indicate promising findings for the potential of technology to promote interaction in a way that relieves strain on the caregiver, enhances relationships and engages people with dementia in social activities. Rigorous investigation using standard, comparable measurements is needed to demonstrate the effects of technological solutions, as well as to explore and address barriers and potential adverse outcomes.

**Supplementary Information:**

The online version contains supplementary material available at 10.1186/s12877-021-02105-0.

## Background

There are currently more than 46 million people worldwide living with dementia, a number that is predicted to triple by 2050 [1]. Dementia is a brain condition that affects cognitive functions, with symptoms including challenges in communication skills and impaired memory function [2]. Holding and maintaining conversations can become increasingly difficult as the disease progresses, resulting in great frustration for both the person with impaired communication capabilities and the conversation partner [3]. Friction in the caregiving dyad may contribute to deterioration of functioning of the person with dementia. In addition, low ratings of relationship quality by people with dementia (PwD) are associated with depression and lowered quality of life (QoL). Communication challenges also affect caregivers, with low ratings of relationship quality associated with greater carer stress [4] and adversely influencing carer wellbeing [3].

Although it is unquestionably crucial to understand the pathology and manifestation of dementia, there is a push for shifting the focus from symptoms and disability towards the capacity and potential of PwD [4]. These capacities can be addressed by the concept of social health, an umbrella term operationalized by the INTERDEM Social Health Taskforce to formulate directions for research and practice to promote social health in dementia. By focusing on enhancing social health, a more balanced view of dementia is promoted, enabling affected individuals to adapt and live well with the changes the condition brings into their lives [4]. This is supported by evidence indicating that maintaining a good dyadic relationship can potentially enhance social health and QoL, slow the progression of functional and cognitive decline, as well as delay institutionalization [5–7]. Social health and good communication in an institutional context is also of upmost importance, as there is evidence to suggest that social interaction is associated with higher QoL amongst PwD residing in long-term care (LTC) facilities [8]. However, the need of PwD for social contact in institutional settings is often unmet. One offered explanation for such a lack of social contact is communication challenges between nursing staff and residents with dementia [8, 9].

Developing technology-based interventions has shown promise for only engaging PwD in meaningful activities, with positive impacts for interaction and social participation. Such interventions also help formal caregivers by providing tools they can use to activate the residents in their care [10]. However, several studies report issues related to usability and user-friendliness of technologies intended for use in dementia caregiving [11–13]. Reported challenges with use of systems designed for this target group relate to complexity [11], technological stability [12], motivation [14] and level of impairment [15]. As a result, it is recommended that the needs of users in a dementia caregiving context be considered on a case-by-case basis to ensure the successful uptake of technology [13].

Despite reported issues, there are also studies showing successful implementation of novel technology for PwD, given that sufficient support is provided from formal or informal caregivers [12, 13, 16, 17]. Considering communication is a collaborative process [18], it is expected that technology-driven interventions can have a mutual influence on both members of the caregiving dyad. This review therefore takes on a dyadic approach, as supporting dyads as an entity has shown to be more effective than focusing on single actors [5]. Although important aspects of communication have been studied, to our knowledge, none of the current reviews focused specifically on studies of technology that prompt conversation and facilitate interaction to enhance the quality of dyadic relationships in a dementia caregiving context. Evidence is scattered on this subject, and a systematic approach is required to synthesize the current body of literature.

### Objective and research question

The aim of this review is to provide a comprehensive description of technology-driven interventions for PwD and their conversation partners to enhance communication and the quality of their relationship. By focusing on “conversation support” – an area of social health in dementia caregiving which at first glance might appear intangible – this review makes a unique contribution to an area that has so far mostly remained in the background of dementia research.

The review is guided by the following research questions:

1. What technology-driven solutions are used to prompt conversation, facilitate communication, and enhance social interaction between PwD and their conversation partner?
2. How do the technological devices aid in achieving these outcomes for both members of the caregiving dyad?
3. What methodologies are utilized to evaluate the effectiveness of these technologies?

## Methods

### Data sources

A systematic review [19] of the literature was conducted on technology-driven interventions for PwD and their conversation partners to enhance communication and facilitate positive social interactions. Three electronic databases were accessed, selected due to their relevance for the scope of this review, including PubMed, PsycINFO, and CINAHL. All records were examined by title and abstract.

### Search terms

Table 1 outlines search terms, including truncation symbols (denoted by *) and Boolean operators (AND, OR). Search terms were chosen to describe the health condition, technology, targeted outcome measures as specified in R1 and interaction setting included in this review, where each term was adapted to the respective format of each database.

**Table 1:** Search terms and results from databases (search completed)

Database	PubMed	CINAHL	PsycInfo
Search 1	(dementia OR Alzheimer* OR “mild cognitive impairment”)		
Results	118,802	35,476	48,516
Search 2	(techno* OR digital* OR tablet OR touchscreen OR computer OR smart OR robot* OR intelligent OR machine OR gerontotechnology)		
Results	489,608	119,421	106,365
Search 3	(engag* OR social* OR communicat* OR convers* OR relationship OR “relationship quality” OR interact* OR participat* OR inclusion OR mood OR affect)		
Results	2,601,023	635,038	829,585
Search 4	(dyad* OR spouse OR family OR relative OR caregiver)		
Results	734,921	213,131	195,005
Search 5	#1 AND #2 AND #3 AND #4		
Results	415	289	139

**Fig. 1:**
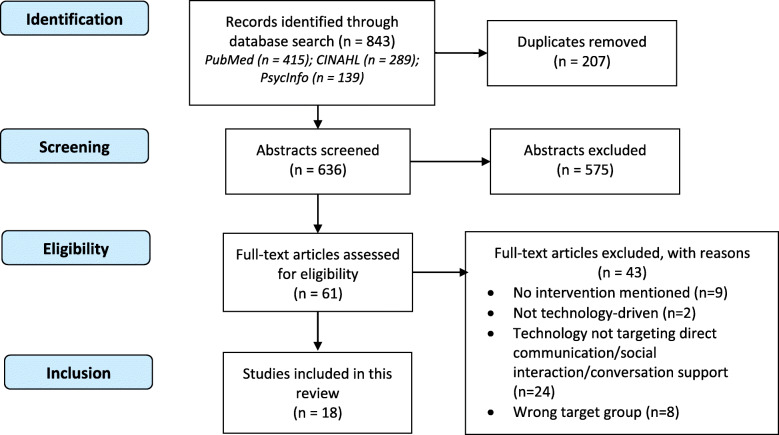
Flow diagram of the study selection process

### Study selection

As there is limited research to address research question 1 (R1), the search for relevant research included studies that did not identify these social and communication measures as the primary outcome. By including papers that reported on secondary findings of enhanced communication, better conversations and improved social interactions, the review explored solutions used to improve these important aspects of social health. As this is a rapidly changing field, only articles published between 2010 and 2019 were included. Papers published in another language than English were excluded from this review.

The search and screening process was undertaken by two researchers working independently (VH and CM). Although disagreement about inclusion was to be settled by a third-party participant (KWO), the initial researchers agreed which papers to include without the need of a third-party settlement. The literature search was conducted between February and March 2020 by VH and in May 2020 by CM.

### Eligibility criteria

During screening, searches of PubMed, CINAHL and PsycINFO generated 415, 289 and 139 papers, respectively (see PRISMA flow diagram in Fig. 1). Records were included or excluded according to the following criteria:

1. Studies describe an intervention. Studies without an intervention component (e.g. only workshops or reviews) were excluded.
2. Interventions identify PwD as the primary target group.
3. Interventions are based on some form of technology.
4. The technology-driven solutions are focused on social interaction between PwD and their conversation partner (i.e. studies describing training technique programs, online support groups or pure monitoring systems were excluded).
5. The technology has some function as conversation aid for interacting, either intended or as a consequence of using the technology.
6. Communication technology is not intended for simulated presence (such as telepresence or a digital conversation partner), as remote interaction is outside the scope of this review.

### Data extraction

Data regarding citation details, technological devices, study design, outcome measures, measurement instruments and major findings was extracted to gain a comprehensive understanding of available evidence in this area. In addition, the quality of the methodology used in the studies was assessed by two researchers (VH and CM) independently, utilizing the Mixed Methods Appraisal Tools (MMAT) checklist [20]. The MMAT score is a rough assessment of methodological quality of the study. By being applicable to both qualitative, quantitative and mixed-method studies, it can be used to appraise the quality of different categories of studies that otherwise would not be comparable [21]. As the review included studies of different designs, the MMAT checklist could therefore be used for all appraisals.

## Results

After exclusion of papers based on examining title and abstracts, duplications were removed, and the remaining 61 papers were read in full. Based on the exclusion criteria, 18 papers were eligible for this review. Additional file 1: Table S2 provides an overview of the studies’ participants, intervention setting and intended use of the evaluated technology. Additional file 2: Table S3 displays a summary of all studies included in this review, including design, technical devices, outcome measures, measurement instruments, main findings relevant for R1 and the quality appraisal score using the MMAT checklist.

Presentation of the results will be guided by the research questions. First, existing technology-driven solutions to support communication and enhance social interaction will be described. Thereafter, the effect of these devices in terms of the outcomes described in R1 will be outlined. Finally, the methodologies of the studies included in this review will be explored. We will report on the interventions following the four identified categories (below) of the technologies used.

### Technological devices to support social interactions

A wide range of devices had reported effects on social interaction either as a primary or secondary outcome. The majority of identified interventions utilized *tablet computers* (*n* = 7) to prompt conversation through applications specifically developed for people living with dementia. Interventions employing *social robots* (*n* = 5) were also reported to facilitate communication between PwD and their caregivers. In addition, *computer systems* (*n* = 4) were identified as beneficial for supporting conversation, either directly or indirectly through engagement in activities. Technology-driven devices not fitting into the three other categories were labelled as *other* (*n* = 2).

#### Tablet computers

Reminiscence activities were the basis of the intervention used for all the tablet-based devices included in this review, with all but one utilizing *personalized* digital applications [22–27]. All tablet-based interventions involved dyadic interaction with the tablet as focal point, either together with staff or visiting family in nursing facilities [22, 24–26] or with spouses in a home-based environment [23, 27]. The frequency and duration of the intervention was rarely specified, and only two studies compared the intervention with a control condition [22, 23].

*Memory Keeper* [26] and *InspireD* [27] were identified as specialized applications containing activity content such as photographs, music and videos helpful to prompt reminiscence and social engagement. Gilson and colleagues evaluated a *collection* of such tailored applications with the same goal [25]. Although each had slightly different software and activation content, all applications were tailored to PwD to facilitate social interaction with staff [25, 26] or at home with their family caregiver [27].

Tyack et al. demonstrated that not all tablet-based reminiscence activities need to be personalized in order to engage PwD and their caregiver in meaningful activities [28]. In this study, a generic art-viewing application was trialed in a home-based setting, targeting enhance well-being for both members of the dyad, as well as the dyadic relationship in itself. The participants were provided with a pre-configured tablet at home, together with a list of sample discussion questions to help generate conversations.

While the above studies observed that conversation was supported through the reminiscence activities, other studies targeted this outcome specifically [22–24]. In one study, an individualized photo- and music database was created as a resource to alleviate the burden of caregivers while visiting their spouses in a nursing home. The visiting spouses were asked to use the tablets for at least 20 min, guided by a music therapist with the goal of prompting communication opportunities [24].

A similar application was evaluated in a single-case study by Ekström and colleagues to explore possibilities and pitfalls of using personalized communication applications to support communication in dementia caregiving [23]. In a home-based setting, a married couple recorded themselves interacting with and without the support of the tabled application *GoTalk NOW*. The application was developed for people with communicative problems, with videos, personal pictures and digitalised and synthetic speech to create individual “communication books” [23].

Finally, social interaction with and without digital support was evaluated by Samuelsson and Ekström in another study included in this review [22]. The researchers investigated the use of two tablet applications, *CIRCUS* and *CIRCA*, for support of dyadic communication between nursing home residents and staff, as compared with interaction without tablets. *CIRCA* was developed as a “cognitive prothesis” [3] to support and promote communication between PwD and their caregivers using reminiscence [22]. *CIRCUS* involved similar reminiscence activities, although offered more individualized experiences as the application can be populated with personal films and pictures.

#### Social robots

The only humanoid robot included in this review was evaluated across three different sites in an international collaboration project; described as a novel technology integrating several capabilities into a single platform, *MARIO* was designed to support several applications supporting cognitive stimulation, social engagement and health assessment [29]. Across all three locations (a nursing home in Ireland, a hospital in Italy and in the community in the United Kingdom), the participants interacted with *MARIO* independently in supervised sessions and were assessed in pre- and post-*MARIO* interactions.

Several studies evaluated the same pet-robot technology, *PARO*, in either home-based or institutional settings [30–32]. This therapeutic robot companion was previously assessed in a multitude of studies for its impact on several social health dimensions [33–36]. Designed as a soft baby harp seal, the social robot is equipped with sensors to perceive its environment and provide physical interaction through sensory information such as vibration and visual and auditory feedback. Moyle et al. [32] and Liang et al. [31] both trialed the social robot in a randomized controlled trial, while Robinson et al. [30] compared *PARO* to another robot, *Guide*, in a cross-sectional study. Although *Guide* is designed for elderly populations without cognitive impairments, it has a wide array of functionalities for entertainment, telephone calling, and checking vital signs [30]. Like the *MARIO*-intervention, participants in all three studies interacted with the robots in a supervised, but unstructured format to allow flexible interactions and explorations - regardless of whether in group- or individual sessions.

In a Swedish study, an interactive robotic cat *JustoCat* was employed to facilitate reminiscence therapy. The researchers assumed that a seal would not appeal to the participants as few had associations with seals [37]. A structured procedure was followed for the intervention, with nursing home caregivers interacting with residents in activities of discovery, engagement and emotional response, touching, petting and holding the robotic cat, while consistently talking about the robot and asking what the participants experienced [37].

#### Computer systems

Although not as intuitive or simple as tablet-computers, computer-based systems show promise in creating tailored interventions for PwD due to their large power supply and extension opportunities. An example is the computer system *Memory Box*, evaluated by Davison et al. [38] in a randomized, single-blinded crossover study. This system could be adapted to play music and display photographs, movies and messages with a simplified interface to facilitate PwD in accessing data material. The participating LTC facility residents interacted with *Memory Box* independently, while staff and visiting family members were encouraged to support its use.

The additional storage and faster processing of computer systems offer many possibilities for creating and developing programs, packages and applications to tailor content to the user. The benefits, challenges and influencing factors for PwD using such a system was evaluated by Lazar and colleagues in a longitudinal evaluation study [39] and a single-case study [40]. The commercially available technology contained an assortment of applications to support social interactions, cognitive stimulation, reminiscence and exercise, and was equipped with a joystick, camera and a hand bike. Residents in LTC facilities used the technology in weekly sessions with the researchers, in the facility’s activity room with staff, or in an activity group.

Karlsson et al. [41] demonstrated the many opportunities that exist with computer systems, by equipping a personal computer with an integrated touchscreen and specialized software. *SenseCam* automatically took photographs every 2 min, while accompanying PwD throughout their day. With the use of an adapted smartphone to annotate the locations of pictures, a *Digital Photograph Diary* provided snapshots of the day, which later could be viewed at home with family members to narrate the day together.

#### Other

The two technologies classified as ‘other’ differed in terms of familiarity and novelty, but both aimed to create autobiographical reminiscence experiences for PwD and their caregivers. A well-known and low-tech device to enhance psychosocial benefits was the DVD-based *Multimedia Biography* (MB) [42]. By using readily available DVD technology, Damianakis and colleagues evaluated personalized biographic content that could be viewed on a home television to stimulate social interactions between PwD and relatives. The participants were instructed to view the MB on a weekly basis over the course of 6 months, with video recordings and interviews collected at baseline and at three- and six-months follow up sessions.

The final technology included in this review is gaining increasing popularity, but was not previously tested in a dementia caregiving setting [43]. Using 3D printing technology, the objective of the device was to stimulate a positive, autobiographical reminiscence experience for participants in an LTC facility with their formal and informal caregivers. Models of personalized objects were created on a computer, ranging from pets, cars and cabins, and then printed using 3D technology to create objects that participants could use for reminiscence-related activities. Visiting family members and staff used the objects as desired to prompt discussion and communication, enabling participants to touch and feel the objects during interaction sessions.

### The impact on social interaction

By analyzing the impacts of the technological device(s) on interaction and relationships, communication and conversation quality, four major themes were identified: i) breaking the ice; ii) increased interaction; iii) better understanding of the person with dementia; and iv) reduced pressure for the conversation partner. Findings reported in the studies, guided by the research questions of this review have been grouped into domains and reported in the section below.

#### Breaking the ice

Technology-driven solutions in social interactions can serve as a conversational platform for the dyads, opening communication or serving as an ice breaker to initiate dialogue [29, 32, 37, 38]. For example, interventions evaluating the effect of *PARO* found that in addition to improving mood, providing comfort and reducing agitation, the robotic seal provided PwD an opportunity for communication with their relatives, as it facilitated conversations and involvement of the family [30, 32] or formal caregivers [30, 31]. Furthermore, social robots prompted dialogue among participants as well as provided a diversion from usual conversations [32, 37].

#### Facilitating interactions

Utilizing technology to support communication has the potential to increase both the frequency and duration of social interactions [23, 26, 30], and encourage more involvement of caregivers [25, 38, 43], positively influencing the relationship between care recipient and carer [24, 26–28, 39, 41]. Although not robust enough to support a causal effect of tablet computers on the frequency of social interactions, several studies found an increase in dyadic interactions when supported by the social facilitator. The tablet-based social interactions resulted in higher amounts of communicative actions and increased frequency and duration of visits [23, 26]. Family members reported that the convenient tablet-technology provided more enjoyable and meaningful interaction, which supported their relationship [24, 26]. Where technological devices stimulated joint activity for the dyad, this in turn contributed to increased communicative interaction by providing a conversational focal point where they could share experiences [40, 41].

Activation stimuli must elicit engagement to be effective [10], a crucial factor for facilitating interaction, as it can generate further encouragement from caregivers, yield enthusiasm or spark enjoyable discussions [28, 38, 43]. The activities described in the studies encouraged the involvement of relatives and staff in the interventions, supported by the technological devices [38, 43]. Engaging in stimulating activities can create additional positive effects, including significant reductions in symptoms of depression and anxiety, thereby enhancing interactions [37, 38, 41]. By interacting with the technology, residents also spontaneously conversed with each other, resulting in enjoyment, increased interactions and connectedness [30, 41].

#### Better understanding the person with dementia

Reminiscence has beneficial effects for connectivity between PwD and their family and friends. Having an aid for communication and stimulus to share memories is an important factor for strengthening connectedness [44]. Digital devices targeting reminiscence therapy in this review consolidated this finding [26, 28, 37, 42]. Technological devices that triggered early memories with PwD allowed family members to gain insight into the lives of their relatives, which frequently led to enhanced communication [42, 43]. Family members of the participants were helped to remember and better understand their loved ones living with dementia. Such effects were also reported for staff working in LTC facilities, as technological biographies enhanced the individual’s personhood and provided perspective that might be overlooked because of behavioral problems that often follow a dementia diagnosis [42].

#### Reduced pressure for the conversation partner

As dementia progresses, the cognitive function most impacted, aside from impaired memory, is arguably communication [3, 4]. Despite differing types of relationships among the dyads reviewed in the studies, all informal caregivers were burdened by the progressive decline in communication with their partner. Including technology in dyadic conversations may reduce this burden by making the distribution of communicative responsibilities more symmetric [22, 24]. A reduced burden of upholding conversation can have a beneficial influence on the dyadic relationship, as exemplified by wives visiting their husbands in LTC facilities. They reported that using a tablet while interacting helped them find ways to communicate, alleviating stress and feelings of disconnection during visits [24]. This finding is strengthened by Samuelsson and Ekström [22], who reported that conversation partners felt less pressure to finding new topics to talk about. By circumventing some of the memory issues that often deters conversation for PwD, tablet computers can potentially support PwD to be more active during social interactions [22].

### Study methodology and quality

Of the 18 studies, nine took a mixed-methods approach, three assessed outcomes using only quantitative measurements, and six had a qualitative design. Of the mixed-methods studies, only the study of Gustafsson et al. [37] reached a 100% score when evaluated using the quality checklist. Five of the nine mixed-methods studies were found to have a strong qualitative component, with a weaker quantitative component, resulting in overall lower MMAT score applications [23, 28, 30, 31, 43]. Two mixed-methods studies had a poor integration of the qualitative and quantitative sections, reducing the overall quality score [22, 39]. Two of the three purely quantitative studies scored only 40% on the checklist [25, 29], while all six qualitative studies reached a score of 80% or higher specifically [24, 26, 32, 40–42].

Semi-structured interviews were the most frequently used approach for collecting qualitative data [22, 28, 30, 32, 38, 39, 41–43], with focus groups conducted in only two of all the included studies [26, 43]. Of the standardized, validated measurement tools, results show that there is a multitude of different instruments used in measuring social health outcomes in the context of communication, interaction and relationships. This makes a comparison of results a challenge, as both *what* is measured as well as *how* it is measured differs immensely. Aspects of psychosocial health, such as agitation, depression and neuropsychiatric symptoms were most often measured by standardized instruments such as the Cohen-Mansfield Agitation Inventory scale (*n* = 3 [31, 37, 38]), the Cornell Scale of Depression in Dementia (*n* = 4 [29, 31, 38, 39]) and the Neuropsychiatric Inventory questionnaire (*n* = 2 [29, 31]).

Few of the standardized instruments in the reviewed studies were able to capture the nature of interactions, evaluate conversational quality and assess relationships. The Caregiver Burden Inventory (CBI), was one of the many instruments utilized to evaluate *MARIO* [29]. This scale evaluates different aspects of the dyadic relationship, but does not address communication [45]. The Quality of Carer-Patient Relationship Scale (QCPR), utilized by Lazar et al. [39], scores the level of conflict/criticism and degree of warmth in a dyadic relationship. Using either the CBI and QCPR, however, is deemed appropriate to rate the burden of care and dyadic relationships for family caregivers, but not professionals. The QUALID [37] and QOL-AD [28, 29, 39] are two scales for quality of life in late-stage dementia and Alzheimer’s Disease, respectively [46–49]. Both scales assess some components of interpersonal relationships and interactions, but do not rate aspects of communication [48, 49]. The only standardized instrument used in the studies to specifically assess engagement in PwD during social interactions was the Observational Measurement of Engagement (OME), developed by Cohen-Mansfield et al. [10], in the *MARIO* project [29]. However, this observational scale does not contain a dimension reflecting on the relationship of the interacting dyad.

## Discussion

This literature review explored the diverse range of technology-driven social interventions for PwD and their conversation partners to enhance communication and the quality of their relationship. Tablet computers and social robots are the most frequently explored types of technology for communication enhancement, conversation support and facilitating positive social interactions. By aiding social interactions, technology has the potential to increase both the frequency and duration of social interactions and encourage more involvement of caregivers. When used in reminiscence sessions, providing leisure activities or functioning as a point for joint attention, these devices can stimulate dialogue and reduce the pressure of the conversation partner to maintain the interactions. Commercially available technology or innovative devices can not only create a platform for the dyad to converse – they can also provide caregivers an opportunity to learn more about their care recipients. Considering the dyadic nature of caregiving, technology-driven interventions to enhance dyadic interaction could be beneficial to promote the mutual understanding for both members of the dyadic relationship [50]. Taken together, adjusting technology to the needs and preferences of PwD is a promising intervention strategy to create meaningful interactions and avoid social isolation.

It is unusual that studies included in this review primarily indicated only positive effects regarding the use of technology. Such results may suggest a bias of trials for reporting only positive outcomes. If such a bias exists, the implications of this review might be overestimated. The short intervention duration and general lack of long-term follow-up measures in the included studies may predispose the findings to such a bias. Due to the progressive nature of dementia, it is concerning that so few of the interventions exceeded half a year, or at least were stratified according to the stage of the disease. Rosenberg et al. [15] found that difficulties in using *everyday* technology increases in people with mild cognitive impairment and is accentuated in mild stage dementia. This carries certain implications when attempting to introduce *new* technology in a dementia caregiving context.

Moreover, the wide range of different technological solutions is reflected in the equally diverse methodology of the studies. A need exists for more high-end research to discuss the effectiveness and efficiency of technology-driven in dementia research, particularly as the body of literature regarding conversation facilitation for PwD using technology to support social interactions is scarce. By not only including studies which explicitly target these outcomes as primary endpoints but also studies which report such outcomes as secondary, this review included a diverse assortment of technologies. Although types of benefits gained from technology were possible to synthesize, the studies included in this review were widely diverse in both intervention delivery and outcome measurements. The quantitative research included in this review allows for greater comparability, while the qualitative research is more explorative in nature. Although all the qualitative studies scored high on the MMAT checklist, such research has limitations for generalizability of results. However, the intention of this review is not to create a toolbox of standardized measurements for the outcomes posed in R1, but rather to explore what existing methodologies are utilized.

With all this said, the few existing standardized measurement instruments that aim specifically to measure communication and interaction in dementia caregiving point to a research gap in the body of literature. Although standardized outcome measures for QoL and wellbeing exist, they do not have the required sensitivity to capture the outcome dimensions that are the focus of this review. This indicates a lack of attention to these aspects of interacting and dyadic communication in dementia caregiving, a concerning finding considering how communication abilities are affected as dementia progresses [3, 4].

Despite the lack of standardized questionnaires and observational scales for evaluating conversation, communication and interaction quality, such qualities were explored in the qualitative studies included in this review. As many of the studies were feasibility trials, or used to further develop the technology, this data collection method appears to be appropriate. Although rigorous investigation using comparable measurements is also needed to demonstrate the potential of technological solutions in this field, it is important to acknowledge the crucial role of qualitative data in providing a deeper understanding of findings of quantitative studies. Qualitative research exploring the experience and individual benefits of these technology-driven solutions for PwD and their caregivers has the potential to assist in ensuring the integration of these supportive aids in caregiving practice. Considering that several of the studies included experiences and perceptions of *both* formal and informal caregivers, this method of data collection can be an invaluable source of information for the application of technology designed to enhance dyadic interactions and conversation quality.

When communication fails, social participation and interactions become increasingly difficult, causing people with dementia to become socially isolated and interfering with their ability to contribute to society and maintain relationships [3, 4]. As identified by the INTERDEM Social Health Taskforce and the European Working Group of People with Dementia (EWGPWD), social, as well as cognitive consequences of dementia require attention in order to optimize social health for this population group [4]. This review attempts to contribute to this overarching goal by providing a comprehensive description of technological solutions for the conversational component of social health.

Although interventions targeting these outcomes for PwD using technological solutions remain in their infancy, this review identified several domains in which these solutions might be beneficial for both PwD, their family members and professional caregivers. As found in this review, technology targeting this population group can generate a wide range of benefits for social health and QoL by sparking active engagement, supporting conversations and making the dyads’ roles more symmetrical while communicating. Despite limitations in the body of literature regarding technology-driven interventions in dementia caregiving, findings suggest benefits for the wellbeing of PwD, as well as their caregivers. If we are to look to technology for support in dementia caregiving, however, we must adequately explore and address the barriers that exist in doing so, and potential adverse outcomes that might emerge.

### Limitations

The conclusions drawn from this review are subject to several limitations, including the search strategy. The limited availability of research focusing on a topic as intangible as technology and enhancement of “better conversations” with and for PwD, warranted relatively liberal inclusion criteria for the outcome measures when screening for eligible papers for review. Thus, in order to have a feasible number of papers to screen, a strict search protocol was followed where only research that met all four search strategies (#1 AND #2 AND # 3 AND #4) were included. More articles arguably could have been found if not all four criteria were required to be met in the initial database searches.

Only peer-review journals were included to ensure quality assurance for the papers selected, and the review comprised only articles published in English. This might further limit the review, as important technological innovations might have been missed. On the other hand, the criteria allow for the inclusion of papers reporting on the outcome measures specified in R1 only as secondary or unintended effects. Thus, many of the papers did not report on all aspects that might be considered relevant to the completion of a comprehensive overview of this topic. Combined with the small number of articles identified, this is a limiting factor in applying practical implications of the findings.

The quality appraisal of the included articles must also be considered in the context of certain limitations. As this review comprised of a wide array of different designs and approaches, the MMAT was deemed appropriate to assess the methodological quality of the included papers, as it allows for an appraisal of methodological quality of studies with diverse designs [20]. However, a well-known limit of assessing methodological quality of studies using the MMAT is its reductive limit [51]. For studies using a mixed-methods approach, the overall score does not exceed the lowest score of one of the components. As an example, if the qualitative section receives a 100% score, but the quantitative only receives a 40% score, the overall score will consequently be 40%. A study might thus have a strong, as well as a weaker component, but the overall lower score might signal a “less valuable” study [52].

## Conclusions

The findings show that research in the area communication and interaction in a dementia caregiving context is still in an explorative phase, where a great deal of work in both academia and clinical practice is needed to evaluate usefulness, acceptability and effectiveness of technological devices such as those reviewed in this article. There is a gap in the body of literature when it comes to high-level evidence of the effectiveness and efficiency of technology in supporting social interaction of caregiving dyads in the context of dementia. As the studies included for review generally had small sample sizes, lacked a control group, and had very short intervention durations without follow-up measurement, this generates weak evidence that is not always generalizable. Furthermore, the varying methodology and outcome measures point to a need for further research into development and validation of new assessment tools for positive outcomes in social health. As few standardized instruments exist in dementia research that specifically capture the nature and quality communication and social interactions in dyadic relationships, spearheaded efforts are needed for the development of such tools. As stated by the INTERDEM Social Health Taskforce and EWGPWD, there is a need for research that will provide insight into the consequences of social support, as well as relevant outcome indicators for social participation interventions [4]. Despite the methodological issues noted, it is clear that there are promising findings of the potential of technology in helping caregiving dyads interact with one another in a way that relieved the burden on the caregiver, enhances the dyadic relationship and actively engages the PwD in meaningful social activities.

To our knowledge, this is the first review to describe technology-driven interventions to support communication and social interaction with and for PwD. Despite promising findings, the difficulties in utilizing technology with this target group must not be trivialized. The mere presence of these supportive devices is not sufficient to ensure sustainable benefits for all members of the caregiving dyad. The ability of not only the technology but also the surrounding environment to adapt to the individual needs and preferences of the dyad is of practical significance. This issue is central to person-centred care, which is an increasingly advocated approach in both academia and health care provision to provide high-quality care [53]. More research is warranted to explore the existing challenges of technology-driven interventions for to promote social health among PwD and their caregivers so that ultimately, the support offered is adapted to each individual's capabilities, preferences and needs.

## Supplementary Information

**Additional file 1: Table S2**. It shows the reviewed studies’ participants, intervention setting and intended use of the reviewed technology.

**Additional file 2: Table S3**. It summarizes the included papers evaluated technology, function, study design (with quality appraisal), trial duration, outcome measures, measurement instruments and important findings.

## Data Availability

Data sharing is not applicable to this article as no datasets were generated or analyzed during the current study.

## Abbreviations

AD: Alzheimer’s Disease
ACE: Addenbrookes’ Cognitive Examination
CBI: Caregiver Burden Inventory
CDT: Clock Drawing Test
CGA: Comprehensive Geriatric Assessment
CMAI: Cohen Mansfield Agitation Inventory
CMAI-SF: Cohen-Mansfield Agitation Scale – Short Form
CSDD: Cornell Scale of Depression in Dementia
EWGPWD: European Working Group of People with Dementia
FAB: Frontal Assessment Battery
LTC: Long-term Care
MCI: Mild Cognitive Impairment
MMSE: Mini-Mental-Stage-Examination
MSPSS: Multidimensional Scale of Perceived Social Support
NPI: Neuropsychiatric Inventory
NPI-Q: Neuropsychiatric Inventory brief Questionnaire
OME: Observational Measurement of Engagement
PAI: Positive Affect Instrument
PwD: People with dementia
QCPR: Quality of Carer-Patient Relationship
QoL: Quality of Life
QoL-AD: Quality of Life in Alzheimer’s Disease
QUALID: Quality of Life in Late-stage Dementia scale
RAID: Rating for Anxiety in Dementia
RS-14: 14-item Resilience Scale
RUD-FOCA: Resource Utilisation in Dementia – Formal Care
SLUMS: Saint Louis University Mental Status Examination
TBA: Tinetti Balance Assessment
WHO-5: World-Health-Organisation-Five Well-Being Index
