# Supplementary material for: Technology-driven solutions to prompt conversation, aid communication and support interaction for people with dementia and their caregivers: a systematic literature review
Source: BMC Geriatr. 2021 Mar 4;21:157. doi: 10.1186/s12877-021-02105-0 (PMC7934553; doi:10.1186/s12877-021-02105-0)
Supplement: Supplementary file 1 — Additional file 1: Table S2. It shows the reviewed studies’ participants, intervention setting and intended use of the reviewed technology. [file 12877_2021_2105_MOESM1_ESM.docx]

**Additional file 1**

Table S2: Participants, intervention setting and intended use of the reviewed technology

| Author(s) (year) | Participants | | | | | Intervention Setting | | | | Intended use | | |
| --- | --- | --- | --- | --- | --- | --- | --- | --- | --- | --- | --- | --- |
|  | PwD | Stage of Dementia | Informal Caregivers | Formal Caregivers | Research staff | Hospital | LTC facility | Day Centre | Home | Group | Dyadic | Independent |
| Damianakis et al. [42] (2010) | n =12 | AD or MCI | n = 27 |  |  |  | X |  | X | X | X |  |
| Dassa [24] (2018) | n = 3 | “middle stage” | n = 3 |  |  |  | X |  |  |  | X |  |
| Davison et al. [38] (2016) | n = 11 | MMSE 16.2 (6-23) | ✓ ^a^ | n = 2 | ✓ |  | X |  |  |  |  | X |
| D'Onofrio et al. [29] (2019) | n = 38 | ? |  | ✓ | ✓ | X | X |  | X |  |  | X |
| Ekström et al. [23] (2017) | n = 1 | ? | n = 1 |  |  |  |  |  | X |  | X |  |
| Garlinghouse et al. [43] (2018) | n = 15 | SLUMS 8.57 | n = 13 | n = 6 |  |  | X |  |  | X | X |  |
| Gilson et al. [25] (2019) | n=1089 | not assessed |  | n = 542 |  |  | X |  |  |  | X |  |
| Gustafsson et al. [37] (2015) | n = 4 | “late stage” | ? | n = 11 |  |  | X |  |  |  | X | X |
| Karlsson et al. [41] (2014) | n = 7 | MMSE 22 (19-25) | n = 7 |  |  |  |  |  | X | X | X |  |
| Laird et al. [27] (2018) | n = 29 | “early to moderate” | n = 29 |  |  |  |  |  | X | X | X |  |
| Lazar et al. [39] (2016) | n = 5 | MMSE 18.5 (16-21) | n = 4 | n = 7 | ✓ |  | X |  |  |  | X | X |
| Lazar et al. [40] (2015) | n = 1 | MMSE 16-21 | n = 1 |  |  |  | X |  |  |  | X | X |
| Liang et al. [31] (2017) | n = 30 | ACE 27.8 | n = 30 |  |  |  |  | X |  |  | X |  |
| McAllister et al. [26] (2017) | n = 3 | ? | n = 6 |  |  |  | X |  |  |  | X |  |
| Moyle et al. [32] (2019) |  | N/A | n = 20 |  |  |  | X |  |  |  |  | X |
| Robinson et al. [30] (2013) | n = 10 | not assessed | n = 11 | n = 5 |  |  | X |  |  |  | X | X |
| Samuelsson & Ekström [22] (2019) | n = 3 | not assessed |  | n = 3 |  |  |  |  | X |  | X |  |
| Tyack et al. [28] (2017) | n = 12 | ? | n = 12 |  |  |  |  |  | X |  | X |  |

“?” *indicates that data was not reported in the study*“✓”*indicates that the number of these particular participants was not relevant for the study* ^a^ *Views of the participants are collected, but the number of participants is not reported
ACE = Addenbrookes’ Cognitive Examination; AD = Alzheimer’s Disease; LTC; Long-term Care; MCI = Mild Cognitive Impairment; MMSE = Mini-Mental-Stage-Examination; PwD = People with Dementia; SLUMS = Saint Louis University Mental Status Examination;*
