# Supplementary material for: Technology-driven solutions to prompt conversation, aid communication and support interaction for people with dementia and their caregivers: a systematic literature review
Source: BMC Geriatr. 2021 Mar 4;21:157. doi: 10.1186/s12877-021-02105-0 (PMC7934553; doi:10.1186/s12877-021-02105-0)
Supplement: Supplementary file 2 — Additional file 2: Table S3. It summarizes the included papers evaluated technology, function, study design (with quality appraisal), trial duration, outcome measures, measurement instruments and important findings. [file 12877_2021_2105_MOESM2_ESM.docx]

Additional file 2: Table S3. Study Characteristics

| *Author(s) (year)* | Technology | Function | Study Design (MMAT Appraisal) | Trial duration | Outcome measures | Measurement instruments | Interaction and conversation Impact Finding(s) |
| --- | --- | --- | --- | --- | --- | --- | --- |
| *Damianakis et al. [42] (2010)* | DVD: Multimedia Biographies (MB) | Reminiscence Therapy | Qualitative observational study with participatory design (Qualitative checklist: 100 % - but small sample size) | 6 months | Experience in developing and viewing the MB; perceived impact of MB on communication between PwD and family, perceived impact of MB on family communication and changes in attitude toward relative with dementia. | Analysis of video recordings of PwD reactions using the technology + semi-structured interviews with PwD and family following at 3- and 6 months post-intervention. | The MB aided relatives in remembering and better understanding their relative with dementia; it stimulated social interactions between PwD and with both family and formal caregivers. The technological solution helped families to gain insights into their relatives’ lives, frequently leading to enhanced communication. |
| *Dassa [24] (2018)* | Tablet Computer: Individualised photo- and music database | Reduce caregivers' burden during visitation by promoting communication opportunities | Case-Study (Qualitative checklist: 100 %) | 2-3 weeks | Spouses' feelings towards visiting their partner in the nursing home + perceived influence of the personalised database on the relationship | Systematic qualitative content analysis of all written reports documenting the process: (Pre-post intervention interview reports, preparation meeting reports, spouses’ recorded reactions at the end of each visit with the PwD + music therapists written log). | All participants reported the that the device supported them in finding ways to communicate with their partner; revive past experiences together; and reduce stress and feelings of disconnection during visitations. |
| *Davison et al. [38] (2016)* | Personal Computer with touch Screen and Monitor: Memory Box | Leisure activities | Mixed Methods Randomised, single-blinded Crossover trial (Qualitative checklist: 60 %) | 4 weeks | Agitation, Depression, Anxiety of PwD, and Treatment Satisfaction, Feasibility and Acceptability of the device as evaluated by participants, staff and family | CMAI*; CSDD*; RAID*; Semi-structured interviews | The device sparked enjoyable discussions with family members during visits as well as with staff members. Some (in)formal caregivers perceived a potential for the device to enhance interactions. Significant reductions in depressive and anxiety symptoms during the course of the intervention. |
| *D'Onofrio et al. [29] (2019)* | Humanoid Robot: MARIO | Multiple functions: Cognitive stimulation, Social Engagement, Health Assessment | Cross-Sectional International Experimental Pilot study, encompassing three different sites (Quantitative checklist: 40 % - low completion of intervention sessions, small sample size at each site, where each group are not directly comparable) | Not reported | Observational Measurement of Engagement; Affect, Resilience, QoL, and Social Aspects | CDT*; FAB*; NPI*; CSDD; MSPSS*; RS-14*; QOL-AD*; CBI*; TBA*; CGA* + OME, Systematic interview, clinical evaluation and review of records in Pre- and Post-MARIO interaction | MARIO was found to facilitate conversations and social engagement with staff and relatives and provided participants the opportunity to talk about their own life. |
| *Ekström et al. [23] (2017)* | Tablet Computer: Personalised Communication Books | Conversation support for PwD and their conversational partners | Mixed Methods Single-Case study with both analysis of qualitative and quantitative characters of recorded activities (Quantitative checklist: 60 % only one subject only) | 2 weeks | Number of communication sessions recorded; length of each session; communicative initiatives made by the PwD; conversational domains; conversational time distributed between dyad | Video recordings analysed focusing on the outcome dimensions, where the differences between the two data sets (recordings with and without tablet) have been in focus | Increased number of communicative actions and amount of interaction with use of communication support. Implication of device encouraging communication for PwD and their conversational partner, but the not strong enough evidence to support this. |
| *Garlinghouse et al. [43] (2018)* | 3D printer printing personal, symbolically significant objects to PwD | To stimulate a positive, autobiographical reminiscence experience for PwD and their (in)formal caregivers | Mixed Methods Feasibility Study with a Parallel Convergent Design (Quantitative checklist: 40 % - homogeneous sample, low completion of intervention, no control condition) | 2 weeks | Feasibility, Utility, Benefits, Engagement, Reminiscence Process | Intervention-specific review checklist (feasibility and utility), Observational notes, Semi-Structured Interviews and Focus Groups | Strong indications that PwD enjoyed using the 3D objects, were engaged while doing so, and seemed to value the objects due to their personalized nature. This also appeared to encourage family involvement as well as increased interactions with residents for both staff and family. The prompts and encouragements offered by family members and staff during reminiscence sessions were further facilitated by the presence of the objects. |
| *Gilson et al. [25] (2019)* | Tablet Computer: with a multitude of apps based on the preferences of the care recipient | Improve socialization, personalized care, and mood management to make the day better for caregivers | Pre-Post quantitative naturalistic Pilot intervention study (Quantitative checklist: 40 %) | Not reported | Mood changes + session influence on caregiver’s daily activities in addition to record the type of engagement activities and whether it was successful or challenging. Variables were thereafter converted into categories of methods for the dyadic engagement (e.g. communication) and type of strategy of the dyad (e.g. socialising). | Proxy reported changes in Mood pre- and post-tablet session by a Visual Analog Mood Scale (VAMS). A subsample of caregivers reported on impact of the session on their day, using a 5-point scale. | For the vast majority, mood was either maintained or enhanced, or was achieved after beginning the session in a negative mood. Socialization, when used as a component of additional strategies together with the tablet session, was also related to PwD having positive mood changes (P=.004). In addition, findings support other research suggesting that gaming apps are not as useful for promoting engagement as apps targeting relaxation or telling an individual’s life story for PwD. Largest effect on caregivers’ day was observed when care recipient's mood was considered to have improved following the session. |
| *Gustafsson et al. [37] (2015)* | Social Robot: Robotic Cat | Reminiscence Therapy | Mixed Methods Pilot Study conducted in two stages: a quantitative Single-case study and a Qualitative interview study (MM checklist: 100 %) | 7 weeks | Single-Case: Agitation and Quality of Life. Qualitative interview stage: Views on interaction, communication, and usage | CMAI; QUALID*; Qualitative Interviews | Quantitative Single Case Stage: Results indicated less agitated behaviour and better QoL. Qualitative interview stage: Formal caregivers and relatives experienced the robotic cat as an opening to communication in caring, breaking the "vicious" circle of constant repetitive behaviour. They also indicated positive effects of the Robotic Cat in providing increased interaction, communication, stimulation and comfort for the PwD |
| *Karlsson et al. [41] (2014)* | Wearable SenseCam, Adapted Smartphone and Personal Computer: Digital Photograph Diary | Remembrance of and conversations about daily life events. | Explorative multiple Case-Study (Qualitative checklist: 100 % - but high drop-out rate) | 6 months | Expectations, Ability to learn and understand, Actual Use, Support, and Experienced Usefulness | Semi-structured interviews | The technology contributed to increased communication and promoted the relationship between family members and the PwD. Usage was perceived as a stimulating joint activity by the individuals with dementia and their family |
| *Laird et al. [27] (2018)* | Tablet Computer | Personalised Tablet Reminiscence | Quasi-Experimental Feasibility Study (Quantitative checklist: 100 %) | 12 weeks | Mutuality, Emotional Wellbeing, and Quality of Carer-Patient Relationship | Mutuality Scale; QCPR*; WHO-5* | Statistically significant increases in Mutuality, Quality of carer and patient relationship, and emotional Wellbeing of PwD from baseline to endpoint. Same (nonsignificant) effects were found for the informal carers. |
| *Lazar et al. [39] (2016)* | Computer System specifically designed for older adults in the community (including a touch-screen monitor) | Active engagement, social interactions and stimulating leisure activities | Mixed Method Longitudinal feasibility study (MM checklist: 20 % - quantitative measurements were just to provide rather a full picture of the residents, not evidence of effectiveness | 6 months | Quantitative: Cognition; Quality of Life; Depression; Resource utilization in dementia care; positive affect, and at baseline: Positive affect instrument regarding relationship with PwD. Qualitative interviews: Perception of Challenges; Benefits; Influencers | QOL-AD; CSDD; RUD-FOCA*; PAI* + Semi-structured interviews | Staff and family members reported benefits for residents such as mental stimulation, enjoyment, interactions and connections with others. The technical system elicited responses to the staff when using the system, in addition to residents conversing spontaneously with each other. Benefits for both staff and family members were reported as well, e.g. better interactions and learning more about residents |
| *Lazar et al. [40] (2015)* | Computer System specifically designed for older adults in the community (including a touch-screen monitor) | Active engagement, social interactions and stimulating leisure activities | Single-Case study of one dyad participating in a larger intervention (Qualitative checklist: 100 %) | 6 months | The experiences of a family member of a PwD living in a Memory Unit Clinic, using the technology. | Unstructured In-depth interview | The PwD looked forward to sessions, in part likely due to her daughter’s enthusiasm, and was able to relive some of the positive interaction afterwards by talking to her. The caregiver was able to expose her mother to a variety of beneficial activities by proactively discovering and facilitating opportunities. I’m learning more and more.” The technology exposed the PwD to new activities, and the caregiver appreciated learning more about her mother’s interests so that she could incorporate these activities in her schedule. |
| *Liang et al. [31] (2017)* | Social Robot: PARO | Alleviate depression, actively engage and improve mode | Mixed Methods Pilot cRCT ( (Quantitative checklist: 80 % as blinded allocation, if possible, was unclear) | 6 weeks (follow-up measures after 12 weeks) | Cognition, Agitation, Neuropsychiatric symptoms, and Depressive symptoms (primary outcomes) for PwD, + Observation of behavioural, affective, social responses and physiological indexes. | CMAI-SF*; CSDD; NPI-Q* + observational scores of physical interactions, attention, and communication with PARO | PwD in the intervention group (PARO) showed non-significantly more positive facial expressions, but they also talked more to staff and researchers compared to participants in the control group |
| *McAllister et al. [26] (2017)* | Tablet Computer: Prototype of digital application Memory Keeper (MK) | Stimulate reminiscences and meaningful engagement with formal and informal carers. | Pilot Explorative Case-Study (Qualitative checklist: 80 % - small sample size and risk of exaggerated positive feedback of experience from participants) | 6 months | Perceived barriers/ facilitators for use of MK in LTC; benefits/setbacks of MK in this setting; the potential use and incorporation of MK in LTC facilities | Observations of participants’ reactions and interactions to the intervention; Focus groups and one individual interview | All participants felt using MK provided more enjoyable and meaningful engagement between the PwD and their relatives + supported their relationship. Frequency and duration of visits increased. MK perceived as an easy and convenient way to enhance the shared experiences of PwD and their visitors. |
| *Moyle et al. [32] (2019)* | Social Robot: PARO | Alleviate depression, actively engage and improve mood | Descriptive qualitative study nested within a larger cluster RCT (Qualitative checklist: 100 %) | 10 weeks | Family member's perception of PARO (compared to control plush toy) + their experience in relation to BPSD | Semi-structured interviews | Mostly positive perceptions of PARO, including reduced agitation, improved mood and provided opportunity for communication for their relative, as it facilitated conversations and involvement. |
| *Robinson et al. [30] (2013)* | Social Robots: PARO and Guide | Facilitate communication, stimulate  attention, and help staff care for patients to the best of their abilities | Mixed-Methods Cross-sectional, single session intervention (Quantitative checklist: 60 %) | 1 hour | Duration and nature of interaction (between PwD and Robots and between PwD and relative) + acceptability and suggestions for improvements during in-depth interviews | Semi-structured interview for staff and relatives after the interaction sessions with both robots. Duration and nature of interaction with the robots were video recorded | Residents spoke unprompted to others about the robot more (although non-significantly) when using Guide but spoke significantly more to Paro than the other robot. It was deducted that entertainment would be the main function of a robot as Guide, which was observed to prompt conversations in many interactions. Paro was praised as stimulus to keep people entertained and also have a calming effect when interacting with an upset resident. |
| *Samuelsson & Ekström [22] (2019)* | Tablet Computer: CIRCA and CIRCUS compared | Communication Aid | Mixed methods Multiple Case-study, mixing quantified analysis of video recordings and interviews (MM checklist: 20 % - unclear integration consideration, unfulfilled quality criteria of each tradition of methods involved) | Not reported | Topic transitions (closing and introducing new topics); initiatives; maintaining conversation; and experiences of the conversations | Video recordings of interactions + semi-structured interviews | PwD were more active in conversations when using CIRCUS and CIRCA without. The conversational partners reported that both digital support devices were useful for making the conversations more symmetrical and finding topics to talk about, as the communication aids were useful for stimulating topics and the conversation partners felt less pressure to uphold the conversations |
| *Tyack et al. [28] (2017)* | Tablet Computer: Viewing art | Reminiscence therapy to enhance well-being for the PwD and their informal Caregivers | Mixed Methods study where quantitative data followed a quasi-experimental, within-subjects design. Qualitative data collected during interviews (Quantitative checklist: 80 % - small sample size recruited by self-selection) | 2 weeks | Wellbeing with subdomains of Cognition, Behaviour, Mood and Relationships, with a sub-theme of Effects on dyadic relationships | QoL-AD*; 3 Visual Analogue Subscale measuring appraisal of own happiness, wellness and interestedness; semi-structured interviews | Indications of yielded Wellbeing of the intervention for the dyads. Conversations seemed to have been generally been enjoyable for the dyads. Enthusiasm was reported as using the app gave couples a new shared activity, likely contributing to a more stimulating environment for the PwD and their informal caregiver |

CBI = Caregiver Burden Inventory; CDT = Clock Drawing Test; CGA = Comprehensive Geriatric Assessment; CMAI = Cohen-Mansfield Agitation Scale; CMAI-SF = Cohen-Mansfield Agitation Scale – Short Form; CSDD = Cornell Scale of Depression in Dementia; FAB = Frontal Assessment Battery; MM checklist = Mixed-Methods checklist; MSPSS = Multidimensional Scale of Perceived Social Support; NPI = Neuropsychiatric Inventory; NPI-Q = Neuropsychiatric Inventory brief Questionnaire; OME = Observational Measure of Engagement; PAI = Positive Affect Instrument; QCPR = Quality of Carer-Patient Relationship; QOL-AD = Quality of Life in Alzheimer’s Disease; QUALID = Quality of Life in Late-Stage Dementia Scale; RAID = Rating for Anxiety in Dementia; RS-14 = 14-item Resilience Scale; RUD-FOCA = Resource Utilisation in Dementia – Formal Care; TBA = Tinetti Balance Assessment; WHO-5 = World-Health-Organisation-Five Well-Being Index
